# The Immune Enhancement of Sodium Lauryl Sulfoacetate in Chickens

**DOI:** 10.4061/2010/485060

**Published:** 2010-05-04

**Authors:** DaRong Cheng, ShanYuan Zhu, HuaiChang Sun

**Affiliations:** ^1^College of Veterinary Medicine, Yangzhou University, Yangzhou 225009, China; ^2^Department of Pharmacy, Jiangsu Animal Husbandry and Veterinary College, Taizhou 225300, China

## Abstract

The purpose of this study is to investigate feasibility of sodium lauryl sulfoacetate (SLS) as an immunoadjuvant in chickens. After treating with 62.5, 125, 250, or 500 *μ*g/mL SLS in vitro, lymphocyte proliferation assay of chicken peripheral blood mononuclear cells showed that the OD_570_ values of all experimental groups, as well as Con A-stimulated group, were significantly higher than that of the untreated control group. After injection with 1.0, 2.0, or 4.0 mg/kg of SLS for 3 consecutive days, chickens were vaccinated with an attenuated vaccine against *Newcastle disease virus* (NDV), and the immunoadjuvant effects of SLS were evaluated on the basis of immune organ index, antibody response, and CD_4_^+^/CD_8_^+^ T-cell ratio. The results confirmed that SLS could enhance NDV-specific antibody response and increase CD_4_^+^/CD_8_^+^ T-cell ratio in vivo. Furthermore, SLS could improve NDV-specific antibody response in thiamphenicol-treated chickens. These data indicate that SLS not only can improve humoral immune response but also reverse the immunosuppressive effect of thiamphenicol in chickens.

## 1. Introduction

**Figure 1:**
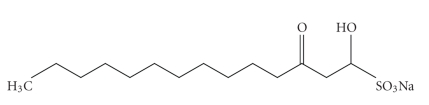
Chemical structure of sodium lauryl sulfoacetate.

*Houttuynia cordata *thunb (Saururaceae, HC) is a therapeutic drug which has been used for the treatment of infections, allergy and cancers [1–9]. The major active component of the herb is houttuynin, which is present in the fraction of volatile oil with antimicrobial, antioxidative, antimutagenic, and immunoadjuvant effects [10–12]. The modified form of houttuynin, called sodium houttuyfonate (SH, C_12_H_23_NaO_5_S, MW 302.36), has potent immunoadjuvant effects including promotion of phagocytosis and secretion of lysozyme, acidic phosphatase and IL-1*β* by macrophages [13–16]. To reduce the cytotoxicitic effect of SH, sodium lauryl sulfoacetate (SLS, brand name is sodium new houttuyfonate, SNH, C_14_H_27_NaO_5_S, MW 330.4, Figure 1) has been generated by additive reaction with houttuynin and sodium bisulfite, which has also been widely used for the treatment of infections, inflammation, anaphylaxis, and cancers [17, 18].

To investigate the feasibility of SLS as an immunoadjuvant for chickens, in this study lymphocyte proliferation assay was performed using chicken peripheral blood mononuclear cells (PBMCs) in the presence of different doses of SLS. Then, the in vivo immunoadjuvant effect was confirmed according to the immune organ index, antibody response, and CD_4_^+^/CD_8_^+^ T-cell ratio of the chickens vaccinated with an attenuated vaccine against *Newcastle disease virus* (NDV) with SLS or SLS plus thiamphenicol treatment.

## 2. Materials and Methods

### 2.1. Materials and Animals

11-day-old unvaccinated local breed Shanhuang chickens were provided by the Jiangsu Institute of Poultry Science. 0.2% SLS in 0.85% sodium chloride solution was provided by the Animal Pharmaceutical Center, Jiangsu Animal Husbandry and Veterinary College. Ficoll-paque lymphocyte separation medium (5.7% (w/v) ficoll 400, 9.0% (w/v) sodium diatrizoate, and D = 1.077 ± 0.002) was from Shanghai Huajing Bio-tech Company Limited. RPMI 1640 medium was the product of GIBCO (USA). Fetal calf serum (FCS) was purchased from Hangzhou Sijiqing Biological Engineering Materials Company Limited. Concanavalin A (Con A), methylthiazolyldiphenyl-tetrazolium MTT bromide, and dimethyl sulfoxide (DMSO) were purchased from Sigma. LaSota NDV vaccine was obtained from QianYuanHao Biological Company Limited. Florfenicol pellets (0.25 g/piece) were purchased from Shanghai Animal Drug Factory. Fluorescein isothiocyanate- (FITC-) labeled monoclonal antibody against chicken CD_4_, phycoerythrin- (PE-) labeled monoclonal antibody against chicken CD_8a_, and biotin-labeled monoclonal antibody against chicken CD_3_ were purchased from Southern Biotech (USA). Streptavidin-allophycocyanin was purchased from Becton Dickinson (USA).

### 2.2. Lymphocyte Proliferation Assay

10 mL of pooled blood sample was collected from 10 chickens and onefold was diluted with PBS. PBMCs were separated by density gradient centrifugation on 14 mL Ficoll-paque lymphocyte separation medium at 400 g for 15 minutes and resuspended (1 × 10^6^ cells/mL) in RPMI 1640 medium supplemented with 10% FCS, 100 IU/mL penicillin, and 100 *μ*g/mL streptomycin [19]. Lymphocyte proliferation was measured using MTT method as described [20]. Briefly, 100 *μ*L of the cell suspension was dispersed into each well of 96-well plates and then 62.5, 125, 250 or 500 *μ*g/mL SLS was added (*n* = 12). After incubation at 39.5°C, 5% CO_2_ for 44 hours, 10 *μ*L MTT (5 mg/mL) was added into each well and the incubation was continued for 4 hours. Then 100 *μ*L of DMSO was added and incubation was continued for additional 24 hours before measurement for OD_570_ values using an ELISA reader (Bio-Tek Instruments, VT). The cell suspension without SLS treatment and with 5 *μ*g/mL Con A was used as the negative and positive control, respectively.

### 2.3. Immunization and Antibody Detection

One hundred forty-four chickens were randomly divided into 4 groups. The three experimental groups were intramuscularly injected with 1, 2, or 4 mg/kg of SLS for 3 consecutive days and the control group was treated in the same way with normal saline. On the third day after injection, all birds were intranasally vaccinated with LaSota NDV vaccine and the immunization was boosted on day 17. On day 10, 17, 24, 31, 38, or 45 after SLS primary treatment, serum samples were collected from all birds and the NDV-specific antibody was titrated using standard hemagglutination inhibition (HI) assay [19].

### 2.4. Determination of CD_4_^+^/CD_8_^+^ T-cell Ratio and Immune Organ Indices

On day 10, 17, 24, 31, 38, or 45 after SLS treatment, 6 chickens of each group were sacrificed and the blood samples were collected for lymphocyte separation. CD_4_^+^/CD_8_^+^ T-cell ratio in PBMCs was measured using flow cytometry [21]. Briefly, 2 × 10^6^ cells from each chicken were incubated at 4°C for 45 minutes with an FITC-labeled monoclonal antibody against chicken CD_4_, a PE-labeled monoclonal antibody against chicken CD_8_, and a biotin-labeled monoclonal antibody against chicken CD_3_. After two washings with PBS, the cells were incubated at 4°C for 30 minutes with streptavidin-allophycocyanin conjugate. After two additional washings, CD_4_^+^/CD_8_^+^ T-cell ratio was determined by flow cytometry. At the end of the experiment, the spleen, thymus, and bursa of Fabricius of each chicken were collected for organ index calculation [22].

### 2.5. Determination of Anti-Immunosuppressive Effect of SLS in Chickens

One hundred and eight chickens were separated into three groups. Each bird in experimental group I was intramuscularly injected with 2.0 mg/kg SLS and then orally administered with 1.2 g/kg thiamphenicol for 3 consecutive days, while the birds in experimental group II or control group were treated in the same way with 1.2 g/kg thiamphenicol or normal saline only. On the third day after treatment, all birds were intranasally immunized with LaSota NDV vaccine and boosted on day 17. On day 10, 17, 24, 31, 38, or 45 after SLS treatment, the serum samples and PBMCs were prepared for determining the NDV-specific antibody response and CD_4_^+^/CD_8_^+^ T-cell ratio as previously described.

### 2.6. Statistical Analysis

The significant difference of the averaged data was analyzed using *t*-test (*P* < .05).

## 3. Results

### 3.1. Enhancement of Lymphocyte Proliferation by SLS

After treatment for 44 hours with 62.5, 125, 250, or 500 *μ*g/mL SLS, the OD_570_ values of all experimental groups were significantly higher than those of the control group, which was comparable to that of Con A-stimulated group (Table 1). Among the four concentrations of SLS tested, the 250 *μ*g group had the highest stimulatory effect on chicken PBMCs, but the difference was not significant. 

### 3.2. Enhancement of Immune Response by SLS

After treatment with different doses of SLS or normal saline, all chickens were vaccinated twice with live NDV vaccine, and serum samples were collected on different days after primary immunization for NDV-specific antibody assay. As Table 2 shows, HI titers of the three experimental groups were 1 or 2 log2 higher than that of the normal saline control group. Among the three doses tested, the 2 mg group had HI titers about 1 log2 higher than those of the other two groups from day 17 after SLS treatment. 

To further investigate the enhancive effect of SLS on antibody response in vaccinated chickens, blood samples were collected on different days after vaccination and CD_4_^+^/CD_8_^+^ T-cell ratios in PBMCs were measured by flow cytometry. As Table 3 shows, all the three experimental groups had significant higher (*P* < .05) CD_4_^+^/CD_8_^+^ T-cell ratios than that of the saline control group from day 31 after SLS treatment. Similar to the antibody response, the 2-mg group has slightly higher CD_4_^+^/CD_8_^+^ T-cell ratio than that of the other two experimental groups.

### 3.3. Influence of SLS on Chicken Immune Organs

On different days after SLS treatment and NDV vaccination, the thymus, spleen, and bursa of Fabricius of each chicken were collected and weighted for organ index calculation. As Table 4 shows, the indices of the three representative immune organs of the three SLS dose groups had no significant differences compared to that of the saline control group.

### 3.4. Anti-Immunosuppressive Effect of SLS in Chickens

After treatment with SLS plus thiamphenicol, thiamphenicol, or normal saline for successive 3 days, all birds were immunized with LaSota NDV vaccine and their serum samples and PBMCs were collected for NDV-specific antibody titration and CD_4_^+^/CD_8_^+^ T-cell ratio determination. As Table 5 shows, HI titer of the SLS plus thiamphenicol-treated group was significantly higher than that of the thiamphenicol-treated group from day 10 after SLS treatment, which was comparable to that of the saline control group. For the CD_4_^+^/CD_8_^+^ T-cell ratio, the SLS-plus-thiamphenicol-treated group had higher score than that of thiamphenicol-treated group from day 10, but significant differences were found only from day 31 after SLS treatment (Table 6). 

## 4. Discussions

Traditional Chinese medicine (TCM) has been widely used for thousands of years, and Chinese scientists have unveiled that many recipes of TCM have played a role in helping to improve the immune system of humans and animals [13]. But it is also facing many challenges; especially, the active ingredients of the most herbs and the role of the active components are still unclear or indistinct. SLS is synthesized artificially by using sodium bisulfite and houttuynin and has been used in the clinic for many years. Recent research has revealed the adjuvant activity and the possible mechanism of SH [13–16]. Whether SLS, the analogue of SH, has a similar effect still needed to be demonstrated.

To this end, in this study the immunoadjuvant effects of SLS were investigated in vitro and in vivo. The results showed that SLS with suitable dose could not only promote the proliferation of chicken PBMCs in vitro but also enhance the NDV-specific antibody response in chickens. The possible reason(s) for this could be due to the increase in chicken CD_4_^+^/CD_8_^+^ T-cell ratio, which indicates the shift of cellular immune response to humoral response [23], since no significant differences in lymphoid organ development were found after SLS treatment. Whether this effect occurs also for other vaccines needs further research.

Thiamphenicol is a popular antibiotic, which is widely used for treatment of bacterial diseases in animals. However, like many other antibiotics, the antibiotic has overt side effects including immunosuppression [24]. Interestingly, the data of this study showed that SLS could promote NDV-specific antibody response in thiamphenicol-treated chickens. Although the detailed mechanism(s) remains to be defined, the experimental data warrant us to further investigate the feasibility of SLS to reverse humoral immunosuppression in other antibiotic(s)-used chickens. This may further widen our knowledge about the role and utilization of TCM in the future.

## Figures and Tables

**Table 1:** Promotion of chicken PBMC proliferation by SLS.

Group (dose)	OD_570 _
SLS (500 *μ*g/mL)	0.154 ± 0.015*
SLS (250 *μ*g/mL)	0.171 ± 0.024*
SLS (125 *μ*g/mL)	0.158 ± 0.017*
SLS (62.5 *μ*g/mL)	0.140 ± 0.031*
Con A (5 *μ*g/mL)	0.149 ± 0.017*
Normal saline	0.073 ± 0.012

The data are expressed as mean ± SD (*n* = 12) in relation to the blank well. *P < .05 was accepted as significant differences compared to the normal saline control.

**Table 2:** The antibody response in SLS-treated and vaccinated chickens.

Group (dose)	HI titer on different days after primary immunization (log2)					
	10 d	17 d	24 d	31 d	38 d
SLS (4 mg/kg)	5.31 ± 0.25	6.02 ± 0.45	6.87 ± 0.27	7.68 ± 0.33*	7.06 ± 0.41	6.53 ± 0.28
SLS (2 mg/kg)	5.72 ± 0.19	6.83 ± 0.33*	7.46 ± 0.49*	8.79 ± 0.11*	8.87 ± 0.52*	8.15 ± 0.19*
SLS (1 mg/kg)	5.11 ± 0.32	6.21 ± 0.42	6.75 ± 0.32	8.02 ± 0.29*	7.46 ± 0.54	6.92 ± 0.16
Normal saline	4.71 ± 0.37	5.12 ± 0.39	5.51 ± 0.37	6.03 ± 0.24	6.14 ± 0.21	5.83 ± 0.31

The data are expressed as mean ± SD (day 10, *n* = 36; day 17, *n* = 30; day 24, *n* = 24; day 31, *n* = 18; day 38, *n* = 12; day 45, *n* = 6) in relation to the HI titers of chickens. *P < .05 was accepted as significant differences compared to the normal saline control group.

**Table 3:** The CD_4_^+^/CD_8_^+^ T-cell ratio in PBMCs of SLS-treated and vaccinated chickens.

Group (dose)	CD_4_^+^/CD_8_^+^ T-cell ratio on different days after primary immunization					
	10 d	17 d	24 d	31 d	38 d
SLS (4 mg/kg)	2.15 ± 0.22	2.31 ± 0.25	2.48 ± 0.22	3.29 ± 0.27*	2.94 ± 0.21*	2.46 ± 0.21
SLS (2 mg/kg)	2.21 ± 0.28	2.49 ± 0.23	2.80 ± 0.17*	3.57 ± 0.31*	3.50 ± 0.19*	3.21 ± 0.24*
SLS (1 mg/kg)	2.13 ± 0.16	2.24 ± 0.22	2.45 ± 0.25	3.41 ± 0.19*	3.10 ± 0.14*	2.47 ± 0.22
Normal saline	2.10 ± 0.23	2.16 ± 0.29	2.19 ± 0.11	2.30 ± 0.23	2.25 ± 0.25	2.11 ± 0.32

The data are expressed as mean ± SD (*n* = 6) in relation to the CD_4_^+^/CD_8_^+^ T-cell ratio of chickens. *P < .05 was accepted as significant differences compared to the normal saline control group.

**Table 4:** Influence of SLS on immune organ indices of NDV-vaccinated chickens.

Organs	Group (dose)	Organ index					
		10 d	17 d	24 d	31 d	38 d	45 d
Thymus	SLS (4 mg/kg)	0.335 ± 0.015	0.351 ± 0.026	0.384 ± 0.021	0.366 ± 0.022	0.360 ± 0.024	0.335 ± 0.015
	SLS (2 mg/kg)	0.327 ± 0.027	0.352 ± 0.023	0.383 ± 0.029	0.368 ± 0.019	0.361 ± 0.021	0.327 ± 0.027
	SLS (1 mg/kg)	0.333 ± 0.028	0.359 ± 0.016	0.380 ± 0.028	0.365 ± 0.031	0.363 ± 0.026	0.333 ± 0.028
	Normal saline	0.328 ± 0.025	0.357 ± 0.025	0.380 ± 0.014	0.364 ± 0.023	0.362 ± 0.024	0.328 ± 0.025

Spleen	SLS (4 mg/kg)	0.163 ± 0.018	0.222 ± 0.025	0.265 ± 0.023	0.270 ± 0.027	0.242 ± 0.020	0.232 ± 0.017
	SLS (2 mg/kg)	0.169 ± 0.019	0.226 ± 0.021	0.270 ± 0.012	0.276 ± 0.018	0.246 ± 0.017	0.269 ± 0.016
	SLS (1 mg/kg)	0.160 ± 0.029	0.223 ± 0.026	0.264 ± 0.019	0.273 ± 0.022	0.243 ± 0.016	0.230 ± 0.019
	Normal saline	0.163 ± 0.016	0.224 ± 0.013	0.266 ± 0.011	0.274 ± 0.027	0.244 ± 0.015	0.234 ± 0.014

Cloacal bursa	SLS (4 mg/kg)	0.331 ± 0.031	0.354 ± 0.027	0.373 ± 0.025	0.355 ± 0.022	0.349 ± 0.021	0.321 ± 0.022
	SLS (2 mg/kg)	0.329 ± 0.030	0.357 ± 0.029	0.369 ± 0.033	0.368 ± 0.028	0.359 ± 0.025	0.325 ± 0.023
	SLS (1 mg/kg)	0.330 ± 0.026	0.355 ± 0.031	0.371 ± 0.037	0.363 ± 0.032	0.357 ± 0.029	0.328 ± 0.024
	Normal saline	0.332 ± 0.019	0.352 ± 0.022	0.370 ± 0.027	0.355 ± 0.028	0.348 ± 0.024	0.331 ± 0.017

The data are expressed as mean ± SD (*n* = 6).

**Table 5:** Anti-immunosuppressive effect of SLS on thiamphenicol.

Group	HI titer on different days after immunization (log2)					
	10 d	17 d	24 d	31 d	38 d
SLS+Florfenicol	4.82 ± 0.29	5.09 ± 0.19	5.46 ± 0.24	6.26 ± 0.19	6.76 ± 0.24	6.43 ± 0.19
Florfenicol	3.01 ± 0.21*	3.43 ± 0.13*	3.83 ± 0.28*	4.06 ± 0.33*	3.96 ± 0.28*	3.83 ± 0.33*
Saline	4.52 ± 0.35	5.05 ± 0.36	5.61 ± 0.34	6.07 ± 0.24	6.21 ± 0.21	5.82 ± 0.31

The data are expressed as mean ± SD (day 10, *n* = 36; day 17, *n* = 30; day 24, *n* = 24; day 31, *n* = 18; day 38, *n* = 12; day 45, *n* = 6) in relation to the HI titers of chickens. *P < .05 was accepted as significant differences compared to the saline control group.

**Table 6:** Influence of SLS on CD_4_^+^/CD_8_^+^ T-cell ratio of thiamphenicol-treated chickens.

Group	CD_4_^+^/CD_8_^+^ T-cell ratio					
	10 d	17 d	24 d	31 d	38 d
SLS+Florfenicol	2.15 ± 0.23	2.24 ± 0.19	2.61 ± 0.17	3.01 ± 0.18*	2.98 ± 0.24*	2.74 ± 0.21*
Florfenicol	2.01 ± 0.19	2.02 ± 0.24	2.02 ± 0.27	2.04 ± 0.23	2.03 ± 0.27	2.00 ± 0.22
Saline	2.09 ± 0.22	2.18 ± 0.27	2.19 ± 0.15	2.30 ± 0.23	2.23 ± 0.24	2.11 ± 0.31

The data are expressed as mean ± SD (*n* = 6) in relation to the CD_4_^+^/CD_8_^+^ T-cell ratio of chickens. *P < .05 was accepted as significant differences compared to the saline control group.
